# Mitochondria-related core genes and TF-miRNA-hub mrDEGs network in breast cancer

**DOI:** 10.1042/BSR20203481

**Published:** 2021-01-27

**Authors:** Li-rong Yan, Ang Wang, Zhi Lv, Yuan Yuan, Qian Xu

**Affiliations:** Tumor Etiology and Screening, Department of Cancer Institute and General Surgery, The First Affiliated Hospital of China Medical University, Key Laboratory of Cancer Etiology and Prevention, China Medical University, Liaoning Provincial Education Department, Shenyang 110001, China

**Keywords:** biomarker, Breast cancer, miRNA, mitochondria, prognosis, transcription factor

## Abstract

Background: Mitochondria-nuclear cross-talk and mitochondrial retrograde regulation are involved in the genesis and development of breast cancer (BC). Therefore, mitochondria can be regarded as a promising target for BC therapeutic strategies. The present study aimed to construct regulatory network and seek the potential biomarkers of BC diagnosis and prognosis as well as the molecular therapeutic targets from the perspective of mitochondrial dysfunction. Methods: The microarray data of mitochondria-related encoding genes in BC cell lines were downloaded from GEO including GSE128610 and GSE72319. GSE128610 was treated as test set and validation sets consisted of GSE72319 and TCGA tissue samples, intending to identify mitochondria-related differentially expressed genes (mrDEGs). We performed enrichment analysis, PPI network, hub mrDEGs and overall survival analysis and constructed transcription factor (TF)-miRNA-hub mrDEGs network. Results: A total of 23 up-regulated and 71 down-regulated mrDEGs were identified and validated in BC cell lines and tissues. Enrichment analyses indicated that mrDEGs were associated with several cancer-related biological processes. Moreover, 9 hub mrDEGs were identified and validated in BC cell lines and tissues. Finally, 5 hub coregulated mrDEGs, 21 miRNAs and 117 TFs were used to construct TF-miRNA-hub mrDEGs network. MYC associated zinc finger protein (MAZ), heparin binding growth factor (HDGF) and Sp2 transcription factor (SP2) regulated 3 hub mrDEGs. Hsa-mir-21-5p, hsa-mir-1-3p, hsa-mir-218-5p, hsa-mir-26a-5p and hsa-mir-335-5p regulated 2 hub mrDEGs. Overall survival analysis suggested that the up-regulation of fibronectin 1 (FN1), as well as the down-regulation of discoidin domain receptor tyrosine kinase 2 (DDR2) correlated with unfavorable prognosis in BC. Conclusion: TF-miRNA-hub mrDEGs had instruction significance for the exploration of BC etiology. The hub mrDEGs such as FN1 and DDR2 were likely to regulate mitochondrial function and be novel biomarkers for BC diagnosis and prognosis as well as the therapeutic targets.

## Introduction

Mitochondria, the only extranuclear organelle carried with genetic material, plays an important role in carcinogenesis through its communication and retrograde regulation of nucleus [1]. The reactive oxygen species in mitochondria were suggested to promote proliferation, migration and apoptosis of tumor cells [2]. The mitochondria in breast cancer (BC) cells could exert retrograde regulation of nucleus by transmitting signal to them, facilitating the bidirectional communication between each other [3] and making mitochondria an anticancer drug target for tumor. In addition, mitochondria from noncancer cell lines has been shown to suppress multiple carcinogenic pathways and reverse the carcinogenic properties of tumor cells under the same nuclear background, including cell proliferation, viability in hypoxia, anti-apoptosis property, resistance to anticancer drugs, invasion, colony formation and enhancing the response of tumor cells to therapy [4]. These findings emphasized that mitochondria had critical regulatory roles in cancer cell property. Some reports showed that mitochondrial-related gene expression might lead to human pathogenesis and the correction with mitochondrial function was a promising target for anticancer therapy [4–6].

MiRNAs could participate in the whole signal pathways of tumor genesis and progression, including the regulation of mitochondrial function [7,8]. Moreover, miRNAs are also involved in the regulation of Otto Warburg effect, thus affecting tumor progression [8]. Transcription factors (TFs) are regulatory factors at transcriptional level for the progression of breast cancer [9,10], and modulate mitochondria biogenesis and mitochondria-to-nuclear communication [11,12]. The transcription of mRNAs and miRNAs are regulated by transcription factors (TFs) and the expression of TFs is modulated by miRNAs [13], both of which are closely related to mitochondria function. Therefore, it is of great importance to construct regulatory network for ‘TF-miRNA-hub mrDEGs’ to explore the mitochondria dysfunction of BC.

Breast cancer (BC) is the most common malignancy and the leading cause of cancer-related death in female [14,15]. The exploration of potential biomarkers and regulatory mechanisms for early diagnosis and therapeutic targets of BC has important scientific significance and application values. In recent years, study remains rare about the differential analysis and network regulation mechanism of mitochondria-related encoding genes in BC. Hence, the model and network construction for predicting early BC diagnosis and prognosis by means of bioinformatics would greatly benefit the identification of potential mitochondrial diagnostic biomarkers, therapeutic targets and pathogenic mechanism for BC. In the present study, two microarray datasets of mitochondria-related genes in BC cell lines were collected from Gene Expression Omnibus (GEO), of which one served as test set and the other served as validation set. Then, the mitochondria-related differentially expressed genes (mrDEGs) were screened out and validated with tissue samples in The Cancer Genome Atlas (TCGA) database. Our study aimed to focus on mrDEGs, construct potential TF-miRNA-mrDEGs network and seek potential diagnostic and prognostic biomarkers as well as the molecular therapeutic targets for BC from the perspective of mitochondrial dysfunction.

## Materials and methods

### Data collection

To identify mrDEGs involved in BC genesis and development, two datasets (GSE128610 and GSE72319) were collected from Gene Expression Omnibus (GEO) (https://www.ncbi.nlm.nih.gov/gds/). GSE128610 contained three BC samples of BC cell lines (MDA-MB-468) and three BC-free samples of BC-free epithelial cell lines (MCF10A). GSE72319 is composed of three BC samples of triple-negative BC cell lines (SUM159) and three BC-free samples of benign BC cell lines (A1N4). Both of them adopted transmitochondrial cybrid system (Cybrid), which was well acknowledged in current mitochondrial function research. For Cybrid model, the nucleus in experimental and control groups were both replaced by other cells’ to eliminate the interference of nuclear encoding genes in mitochondrial function research [16,17]. BC data were downloaded from The Cancer Genome Atlas (TCGA, https://www.cancer.gov/tcga), including 1112 BC tissues and 113 normal breast tissues [18].

### Data processing

In this research, GSE128610 was treated as test set, while GSE72319 and TCGA data respectively served as the first and second validation set. To identify mrDEGs, original microarray datasets of GSE128610 and GSE72319 were analyzed with GEO2R, and TCGA data were processed with edgeR and SangerBox. GSE72319 and TCGA were successively employed to verify mrDEGs in GSE128610 through ‘MATCH function’. The screening and validation criteria were set as |logFC|>= 1, *P*<0.05 and P. adjust<0.05. FunRich 3.1.3 was used to plot the heatmap to visualize the validated mrDEGs in GSE128610.

### GO enrichment analysis and KEGG mapping

Gene ontology (GO) analysis was performed for validated mrDEGs with Search Tool for the Retrieval of Interacting Genes (STRING, https://string-db.org/), including cellular component, molecular function and biological process [19]. *P*<0.05 was considered as statistical significance. KEGG mapper (https://www.genome.jp/kegg/mapper.html) was applied to Kyoto Encyclopedia of Genes and Genomes (KEGG) pathway map of mrDEGs.

### Protein–protein interaction (PPI) network construction and modeling analysis

PPI network of validated mrDEGs was constructed with STRING. The cut-off value of Interaction score was set as 0.4, and PPI network was visualized. Subsequently, classical models were screened out by Molecular Complex Detection (MCODE) plug-in of Cytoscape_3.7.2 based on the criteria of score ≥ 3 and nodes ≥ 3 [20]. The function enrichment analysis of single model was performed by STRING, and *P*<0.05 was regarded as cut-off criteria.

### Screening and tissue identification of hub mrDEGs

Hub mrDEGs were selected by cytohubba plug-in of Cytoscape_3.7.2 according to MCC ≥ 6 [20]. Next, Ualcan (http://ualcan.path.uab.edu/) was utilized to validate the expression levels of mrDEGs in BC tissue samples [21].

### Construction of TF-hub mrDEGs network and miRNA-hub mrDEGs network

NetworkAnalyst (https://www.networkanalyst.ca/), a website for comprehensive gene expression analysis, meta-analysis and network biology, was applied to search TFs and miRNAs targeting hub mrDEGs [22–24]. Hub mrDEGs were uploaded to NetworkAnalyst to acquire the TFs targeting hub mrDEGs from ENCODE database and miRNA-hub mrDEGs pairs from TarBase and miRTarBase. Two interaction lists were downloaded and Cytoscape_3.7.2 was used to visualize TF-hub mrDEGs network and miRNA-hub mrDEGs network respectively.

### TF-miRNA-hub mrDEGs network construction and validation

In order to construct the TF-miRNA-hub mrDEGs network, TF-hub mrDEGs network and miRNA-hub mrDEGs network were overlapped by ‘MATCH function’. Coregulated hub mrDEGs of TFs and miRNAs were selected to construct TF-miRNA-hub mrDEGs network and visualized with Cytoscape_3.7.2.

Subsequently, we searched miRNA-hub mrDEGs network in starBase database (http://starbase.sysu.edu.cn/starbase2/mirMrna.php) and TF-hub mrEGs network in ChEA database [25,26] to screen overlapped hub mrDEGs between the two networks and validate TF-miRNA-hub mrDEGs network. The validated TF-miRNA-hub mrDEGs network was also visualized with Cytoscape_3.7.2.

### Survival analysis based on coregulated hub mrDEGs by TFs and miRNAs

The correlation between coregulated hub mrDEGs and the overall survival of 1402 BC cases was analyzed by using the Kaplan–Meier plotter database (www.kmplot.com) [27]. The mRNA gene chip of BC was chosen in this database. All BC patients were divided into two groups according to the median of gene expression to perform survival curve analysis. The hazard ratios with 95% confidence intervals and *P* values of log-rank test were calculated and displayed in the figure. *P*<0.05 was considered to be statistically significant.

## Results

### The identification and validation of mrDEGs in BC

**Figure 1:**
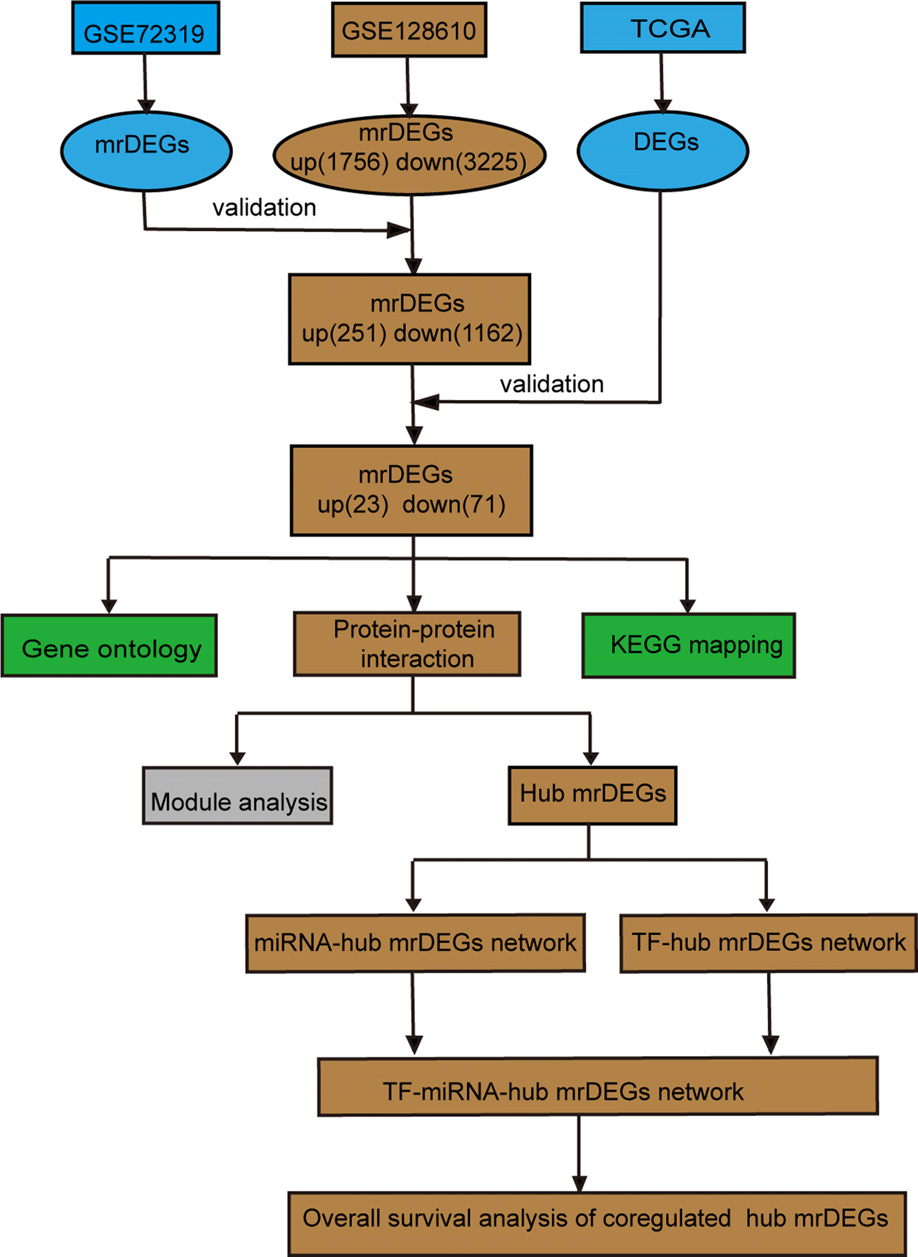
Flow diagram of bioinformatics analysis GSE72319 and GSE128610 were downloaded from GEO database (https://www.ncbi.nlm.nih.gov/gds/); DEGs, differential expressed genes; GEO, Gene Expression Omnibus; KEGG, Kyoto Encyclopedia of Genes and Genomes; mrDEGs, mitochondria-related differential expressed genes; TCGA, The Cancer Genome Atlas, https://www.cancer.gov/tcga; TF, transcription factor.

**Figure 2:**
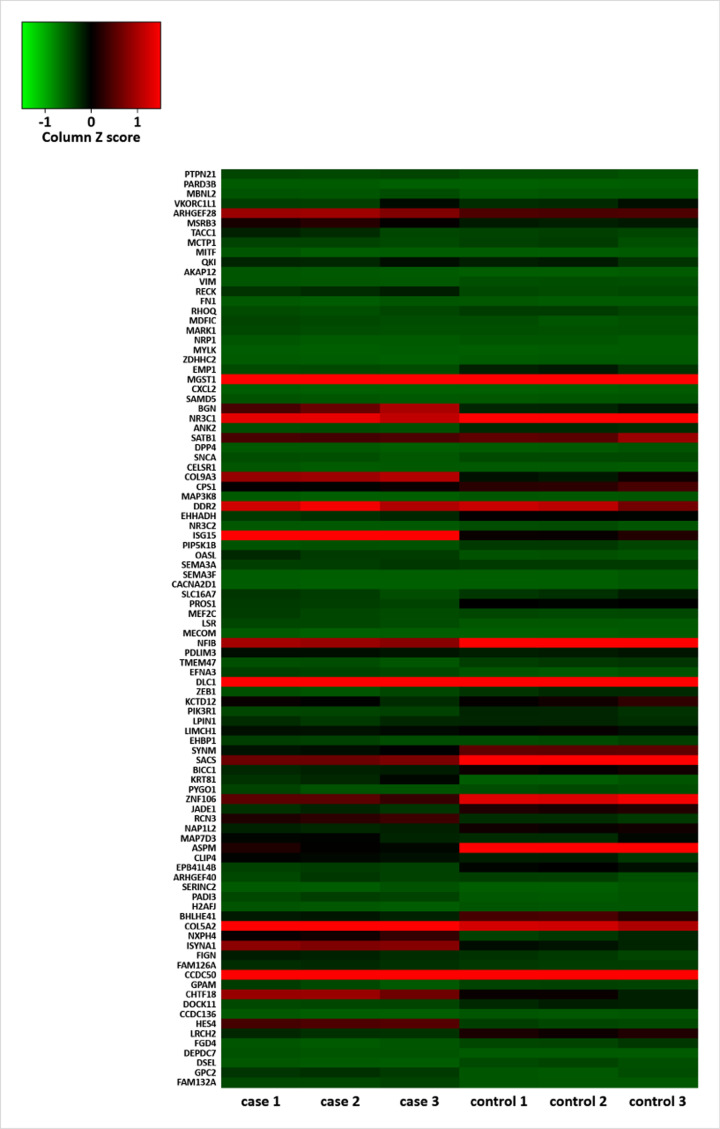
Heatmap of 94 mitochondria-related differential expressed genes in GSE128610 Case 1, case 2 and case 3 were BC cell lines (MDA-MB-468), and control 1, control 2 and control 3 were BC-free epithelial cell lines (MCF10A). Individual values on the vertical axis represented mrDEGs. Green color represented down-regulated mrDEGs and red color represented up-regulated mrDEG; mrDEGs, mitochondria-related differential expressed genes.

The flow diagram was shown in Figure 1. GSE128610 and GSE72319 datasets of BC cell lines were analyzed online with GEO2R. MrDEGs in BC were identified based on the cut-off criteria of |logFC| ≥ 1, *P*<0.05 and P. adjust<0.05. We found out 1756 up-regulated and 3225 down-regulated mrDEGs in GSE128610. Subsequently, 251 up-regulated and 1162 down-regulated mrDEGs in GSE128610 were validated with GSE72319. After initial validation, they were further verified with tissue samples in TCGA database, and then 23 up-regulated and 71 down-regulated mrDEGs were finally identified (Table 1 and Figure 2). The criteria for both validation in GSE72319 and TCGA were the same with GSE128610 (|logFC| ≥ 1, *P*<0.05 and *P*. adjust<0.05).

**Table 1:** Validation of mrDEGs via GSE72319 and TCGA

mrDEGs	Total	Genes name
up-regulated	23	BGN, LSR, SEMA3F, RCN3, PADI3, DEPDC7, OASL, ISG15, H2AFJ, EFNA3, FN1, COL5A2, CHTF18, COL9A3, FAM132A, SERINC2, KRT81, ISYNA1, NXPH4, ASPM, CELSR1, GPC2, HES4
down-regulated	71	NAP1L2, EPB41L4B, ZEB1, MSRB3, QKI, DOCK11, JADE1, SNCA, PROS1, TACC1, CXCL2, CCDC136, RHOQ, MECOM, PIK3R1, PYGO1, SYNM, EHHADH, MEF2C, MDFIC, MBNL2, ZDHHC2, SLC16A7, FGD4, TMEM47, MAP3K8, ANK2, VIM, EHBP1, ARHGEF40, PTPN21, DSEL, DDR2, KCTD12, MITF, CACNA2D1, MGST1, FIGN, SAMD5, NR3C2, FAM126A, VKORC1L1, MYLK, ARHGEF28, AKAP12, CLIP4, ZNF106, CPS1, DLC1, PIP5K1B, LPIN1, BHLHE41, SATB1, MCTP1, NRP1, CCDC50, DPP4, MARK1, SEMA3A, NR3C1, EMP1, SACS, NFIB, LRCH2, LIMCH1, RECK, BICC1, PDLIM3, MAP7D3, PARD3B, GPAM

Abbreviations: mrDEGs, mitochondria-related differential expressed genes; TCGA, The Cancer Genome Atlas.

### GO function enrichment and KEGG pathway map for mrDEGs

GO enrichment analysis for 94 validated mrDEGs was performed by STRING. The results were presented in Table 2 and Supplementary Table S1. The mrDEGs in BC were shown to function in cancer-related biological processes, such as neural crest cell migration involved in autonomic nervous system development (e.g. FN1, semaphorin 3A (SEMA3A), semaphorin 3A (SEMA3F) and neuropilin 1 (NRP1)), regulation of cell migration (e.g. C-X-C motif chemokine ligand 2 (CXCL2), DDR2 and FN1), cell surface receptor signaling pathway (e.g. Microtubule Affinity Regulating Kinase 1 (MARK1), biglycan (BGN) and CXCL2) and cell differentiation (e.g. Ankyrin 2 (ANK2), Glypican 2 (GPC2) and melanocyte inducing transcription factor (MITF)). However, no significant molecular function or cellular component was observed.

**Table 2:** GO enrichment analysis of mitochondria-related differential expressed genes

Term ID	Term description	FDR	Genes
GO:1901166	neural crest cell migration involved in autonomic nervous system development	0.00015	FN1, NRP1, SEMA3A, SEMA3F
GO:0030334	regulation of cell migration	0.0054	CXCL2, DDR2, DLC1, EPB41L4B, FN1, LIMCH1, MCTP1, MEF2C, MYLK, NRP1, PIK3R1, RECK, SEMA3A
GO:0007166	cell surface receptor signaling pathway	0.006	ARHGEF28, BGN, CELSR1, CXCL2, DDR2, EFNA3, FN1, GPC2, ISG15, MAP3K8, MARK1, MEF2C, MITF, NRP1, OASL, PIK3R1, PYGO1, RHOQ, SEMA3A, SEMA3F, SNCA, VIM, ZEB1, ZNF106
GO:0030154	cell differentiation	0.0172	ANK2, ARHGEF28, ASPM, BHLHE41, CCDC136, CELSR1, CPS1, DOCK11, EFNA3, FN1, GPC2, HES4, KRT81, LSR, MARK1, MECOM, MEF2C, MGST1, MITF, NAP1L2, NFIB, NRP1, PIK3R1, PYGO1, QKI, SATB1, SEMA3A, SEMA3F, VIM, ZEB1
GO:0010646	regulation of cell communication	0.0114	AKAP12, ANK2, ARHGEF28, ARHGEF40, ASPM, BGN, BICC1, CXCL2, DEPDC7, DLC1, DPP4, FAM132A, FGD4, FN1, GPC2, JADE1, MAP3K8, MCTP1, MDFIC, MECOM, MEF2C, NRP1, PIK3R1, PIP5K1B, RHOQ, SEMA3A, SEMA3F, SNCA, ZEB1

Abbreviations: FDR, false discovery rate; GO, gene ontology; ID, Identity document.

**Figure 3:**
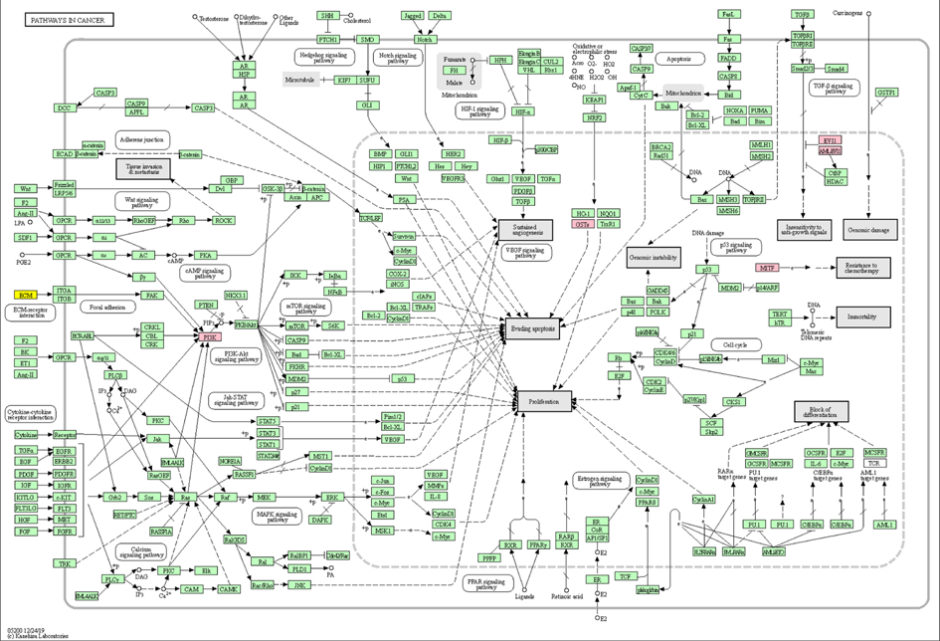
mrDEGs were annotated in KEGG pathway by KEGG mapper (https://www.genome.jp/kegg/mapper.html) Pink color represented that mrDEGs was down-regulated in related-pathway and yellow color represented that mrDEGs was up-regulated in related-pathway; KEGG, Kyoto Encyclopedia of Genes and Genomes; mrDEGs, mitochondria-related differential expressed genes.

The mrDEGs were mapped in KEGG pathway. They were suggested to participate in the following cancer-related pathways: PI3K-AKT pathway (e.g. phosphoinositide-3-kinase regulatory subunit 1 (PIK3R1)), TGF-beta pathway (e.g. MITF), evading apoptosis (e.g. microsomal glutathione S-transferase 1 (MGST1)) and resistance to chemotherapy (e.g. MITF) (Figure 3).

### MrDEGs-related PPI network construction and modeling analysis

**Figure 4:**
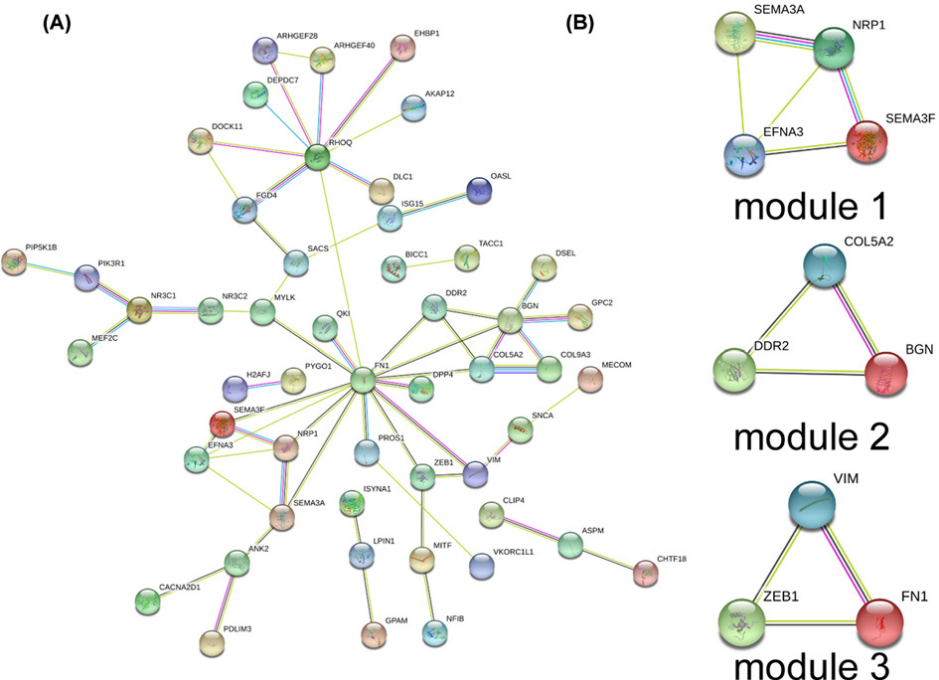
Protein–protein interaction network and top 3 modules of 94 mrDEGs in breast cancer (**A**) PPI network construction by STRING (https://string-db.org/). (**B**) Top modules 1-3 construction by STRING and Cytoscape software; mrDEGs, mitochondria-related differential expressed genes.

PPI network of 94 mrDEGs in BC was constructed by STRING database, with a total of 94 nodes and 60 edges (Figure 4A). Three models were found to meet the criteria of score ≥ 3 and nodes ≥ 3 by MOCODE plug-in of Cytoscape software (Figure 4B). GO and KEGG enrichment analyses were carried out for the three models, respectively (Table 3 and Supplementary Table S2). The results showed that model 1 was mainly associated with the structure and function of nerve cells, model 2 was involved in extracellular matrix and model 3 took parts in tissue development and gene expression.

**Table 3:** Enrichment analysis of models

Category	Term ID	Term description	FDR	Genes
Module 1	GO:0021612	Facial nerve structural organization	3.66E-08	NRP1, SEMA3A, SEMA3F
	GO:0021637	Trigeminal nerve structural organization	3.66E-08	NRP1, SEMA3A, SEMA3F
	GO:0021785	Branchiomotor neuron axon guidance	3.66E-08	NRP1, SEMA3A, SEMA3F
	GO:0038191	Neuropilin binding	9.71E-05	SEMA3A, SEMA3F
	hsa04360	Axon guidance	4.53E-08	EFNA3, NRP1, SEMA3A, SEMA3F
Module 2	GO:0030198	Extracellular matrix organization	0.00093	BGN, COL5A2, DDR2
	GO:0030199	Collagen fibril organization	0.0011	COL5A2, DDR2
	GO:0001503	Ossification	0.0241	COL5A2, DDR2
Module 3	GO:0019221	Cytokine-mediated signaling pathway	0.0121	FN1, VIM, ZEB1
	GO:0045664	Regulation of neuron differentiation	0.0121	FN1, VIM, ZEB1
	GO:0009888	Tissue development	0.0246	FN1, VIM, ZEB1
	GO:0010628	Positive regulation of gene expression	0.0268	FN1, VIM, ZEB1
	GO:0045666	Positive regulation of neuron differentiation	0.027	FN1, ZEB1

Abbreviations: FDR, false discovery rate; GO, gene ontology; ID, Identity document.

### Selection and tissue validation of hub mrDEGs

**Figure 5:**
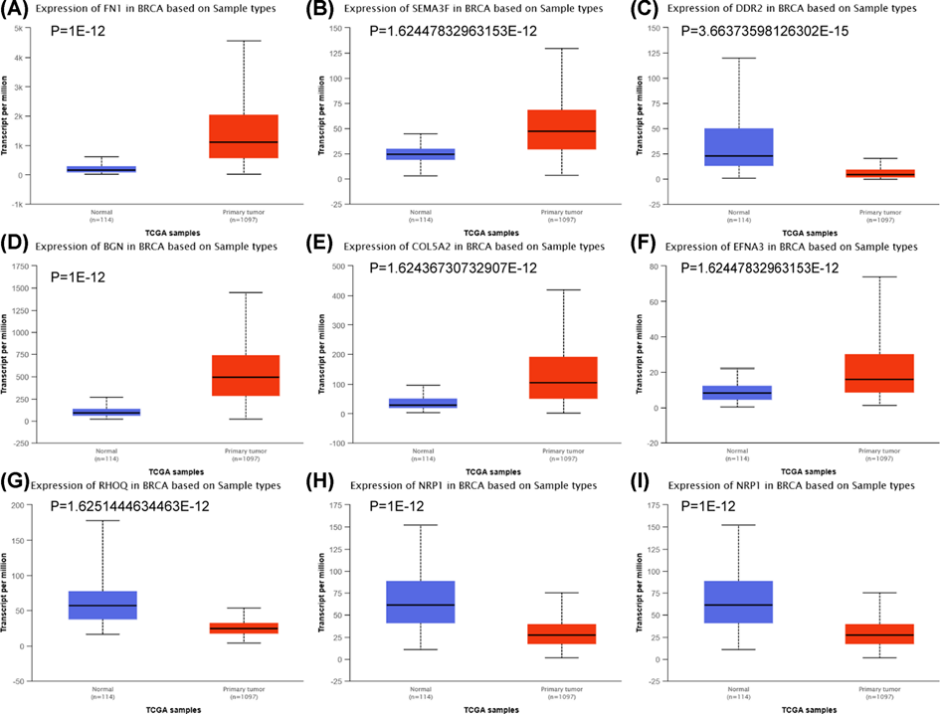
The expression levels of hub mrDEGs in TCGA tissue samples by Ualcan (http://ualcan.path.uab.edu/) Blue color represents normal breast tissues (*n*=114) and red color represents primary breast cancer tissues (*n*=1097). FN1 (**A**), SEMA3F (**B**), DDR2 (**C**), BGN (**D**), COL5A2 (**E**), EFNA3 (**F**), RHOQ (**G**), NRP1 (**H**), SEMA3A (**I**); mrDEGs, mitochondria-related differential expressed genes; TCGA, The Cancer Genome Atlas, https://www.cancer.gov/tcga.

We screened out 9 hub mrDEGs according to MCC ≥ 6 by cytoHubba plug-in of Cytoscape. Among them, up-regulated hub mrDEGs contained FN1, BGN, ephrin A3 (EFNA3), collagen type V alpha 2 chain (COL5A2) and SEMA3F, and down-regulated hub mrDEGs comprised ras homolog family member Q (RHOQ), SEMA3A, NRP1 and DDR2 (Table 4). Consistent results were obtained after validating and visualizing the expression of 9 hub mrDEGs in Ualcan database with tissue samples from TCGA (Figure 5).

**Table 4:** Hub mrDEGs of MCC ≥ 6

Genes name	MCC	Differentially expressed
FN1	25	Up-regulated
EFNA3	12	Up-regulated
NRP1	12	Down-regulated
BGN	10	Up-regulated
RHOQ	9	Down-regulated
COL5A2	8	Up-regulated
SEMA3A	7	Down-regulated
DDR2	6	Down-regulated
SEMA3F	6	Up-regulated

Abbreviations: MCC, Maximal Clique Centrality; mrDEGs, mitochondria-related differential expressed genes.

### Analysis of TF-hub mrDEGs network

**Figure 6:**
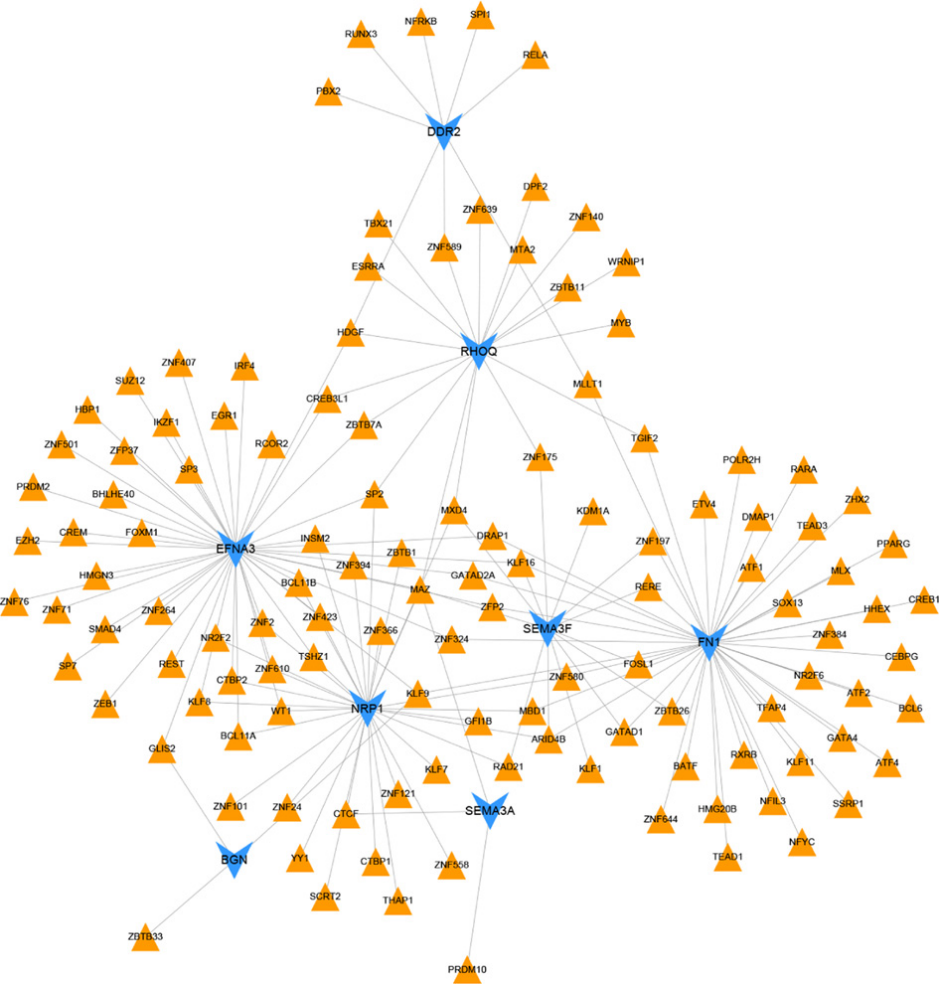
TF-hub mrDEGs interaction network construction by NetworkAnalyst (https://www.networkanalyst.ca/) and Cytoscape software Blue color represented mrDEGs and orange color represented TF. mrDEGs, mitochondria-related differential expressed genes; TF, transcription factor.

In order to explore the potential regulatory relationship of hub mrDEGs, we predicted the TFs targeting hub mrDEGs with ENCODE database. The result demonstrated that 8 hub mrDEGs were matched except for COL5A2. The Cytoscape software was applied to visualize the TF-hub mrDEGs network, and 167 links among 8 hub mrDEGs and 121 TFs were predicted (Figure 6). MAZ could regulate 5 hub mrDEGs (e.g. EFNA3, SEMA3A and SEMA3F). SP2, MAX dimerization protein 4 (MXD4), Kruppel-like factor 9 (KLF9), Kruppel-like factor (KLF16), HDGF and AT-rich interaction domain 4B (ARID4B) regulated 3 hub mrDEGs.

### Analysis of miRNA-hub mrDEGs network

**Figure 7:**
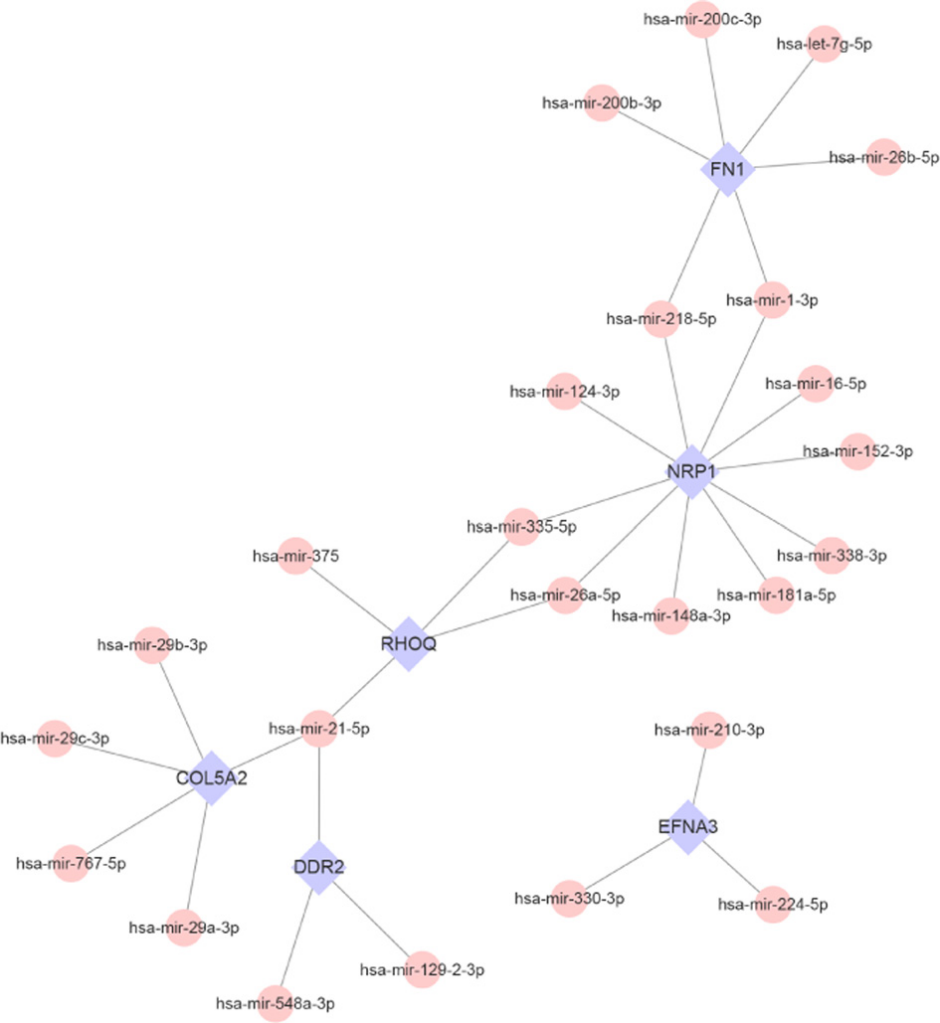
miRNA-hub mrDEGs interaction network construction by NetworkAnalyst (https://www.networkanalyst.ca/) and Cytoscape software Blue color represented mrDEGs and pink color represented miRNA; mrDEGs, mitochondria-related differential expressed genes.

Subsequently, miRNA-hub mrDEGs pairs of 9 hub mrDEGs were performed with TarBase and miRTarBase. Finally, only 6 hub mrDEGs were mapped except for BGN, SEMA3F and SEMA3A, then 31 links among 25 miRNAs and 6 hub mrDEGs were obtained by Cytoscape software (Figure 7). We found that hsa-mir-21-5p could interact with 3 hub mrDEGs including COL5A2, DDR2 and RHOQ.

### Construction and validation of TF-miRNA-hub mrDEGs network analysis

**Figure 8:**
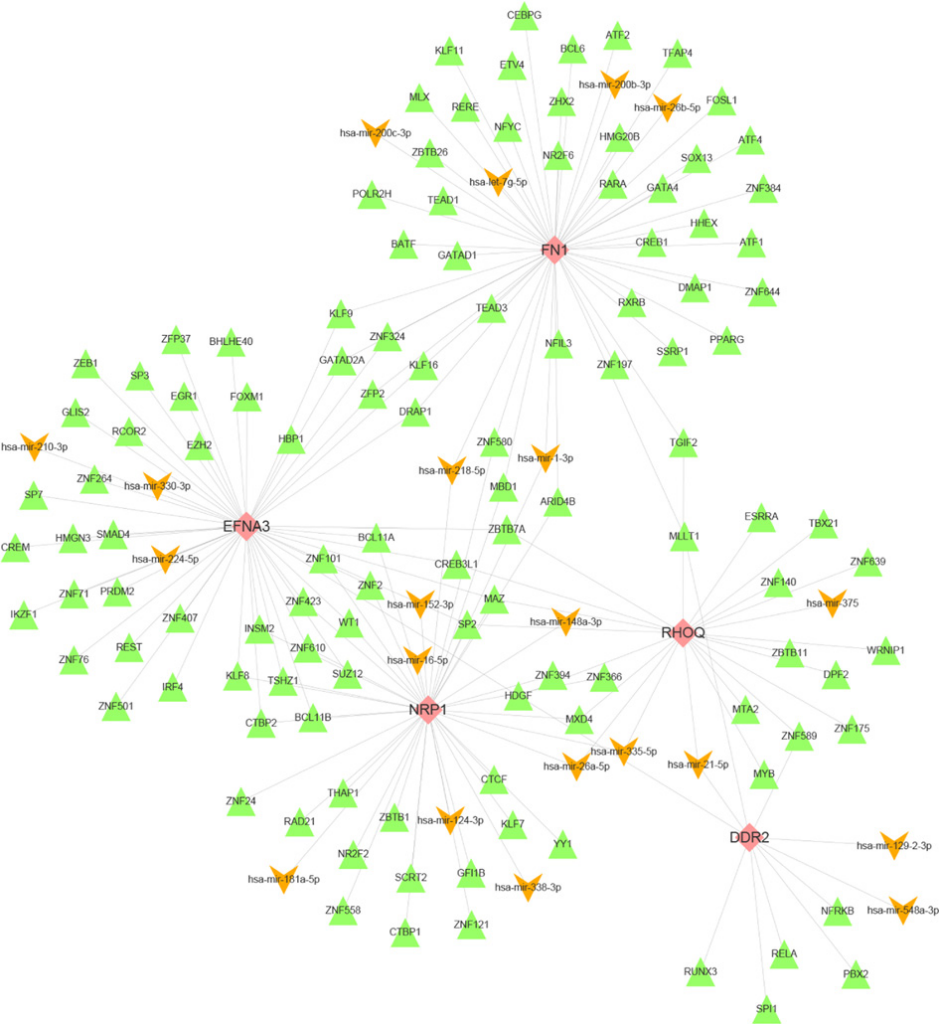
TF-miRNA-hub mrDEGs interaction network construction by NetworkAnalyst (https://www.networkanalyst.ca/) and Cytoscape software Pink color represented mrDEGs, orange color represented miRNA and green color represented TF; mrDEGs, mitochondria-related differential expressed genes; TF, transcription factor.

The hub mrDEGs (FN1, EFNA3, NRP1, RHOQ and DDR2) coregulated by TFs and miRNAs were selected and their interactive regulators were extracted. Then, TF-miRNA-hub mrDEGs network was constructed with Cytoscape (Figure 8). A total of 5 hub mrDEGs, 21 miRNAs and 117 TFs were included in the TF-miRNA-hub mrDEGs network. Next, we analyzed the interactive results of TF-mrDEGs and miRNA-hub network, respectively (Table 5). We found that MAZ, HDGF and SP2 could regulate 3 hub mrDEGs. Simultaneously, hsa-mir-21-5p, hsa-mir-1-3p, hsa-mir-218-5p, hsa-mir-26a-5p and hsa-mir-335-5p regulated 2 hub mrDEGs. In addition, FN1, EFNA3 and NRP1 had the highest degree in the network.

**Table 5:** Hub mrDEGs of coregulated by miRNAs and TFs

TF	Hub mrDEGs	Gene counts	miRNA	Hub mrDEGs	Gene counts
MAZ	EFNA3, NRP1, RHOQ	3	hsa-mir-218-5p	NRP1, FN1	2
HDGF	DDR2, EFNA3, RHOQ	3	hsa-mir-26a-5p	NRP1, FN1	2
SP2	EFNA3, NRP1, RHOQ	3	hsa-mir-335-5p	NRP1, RHOQ	2
ARID4B	FN1, NRP1	2	hsa-mir-1-3p	NRP1, FN1	2
BCL11A	EFNA3, NRP1	2	hsa-mir-21-5p	DDR2, RHOQ	2
BCL11B	EFNA3, NRP1	2	hsa-mir-338-3p	NRP1	1
CREB3L1	EFNA3, RHOQ	2	hsa-mir-16-5p	NRP1	1
CTBP2	EFNA3, NRP1	2	hsa-mir-148a-3p	NRP1	1
DRAP1	EFNA3, FN1	2	hsa-mir-181a-5p	NRP1	1
GATAD2A	EFNA3, FN1	2	hsa-mir-124-3p	NRP1	1
KLF8	EFNA3, NRP1	2	hsa-mir-152-3p	NRP1	1
MBD1	FN1, NRP1	2	hsa-mir-26b-5p	FN1	1
MLLT1	DDR2, FN1	2	hsa-mir-200b-3p	FN1	1
MXD4	NRP1, RHOQ	2	hsa-let-7g-5p	FN1	1
TGIF2	FN1, RHOQ	2	hsa-mir-200c-3p	FN1	1
TSHZ1	EFNA3, NRP1	2	hsa-mir-548a-3p	DDR2	1
WT1	EFNA3, NRP1	2	hsa-mir-129-2-3p	DDR2	1
ZBTB7A	EFNA3, RHOQ	2	hsa-mir-375	RHOQ	1
ZFP2	EFNA3, FN1	2	hsa-mir-210-3p	EFNA3	1
ZNF2	EFNA3, NRP1	2	hsa-mir-224-5p	EFNA3	1
ZNF324	EFNA3, FN1	2	hsa-mir-330-3p	EFNA3	1
ZNF423	EFNA3, NRP1	2			
ZNF580	FN1, NRP1	2			
ZNF589	DDR2, RHOQ	2			
ZNF610	EFNA3, NRP1	2			

Abbreviations: mrDEGs, mitochondria-related differential expressed genes; TFs, transcription factors.

Subsequently, validation of TF-miRNA-hub mrDEGs network was performed by starBase and ChEA. It was shown that 3 hub mrDEGs (FN1, EFNA3 and NRP1), 10 miRNAs and 6 TFs were validated and visualized with Cytoscape software (Supplementary Figure S1).

### Survival analysis of coregulated mrDEGs by TFs and miRNAs

**Figure 9:**
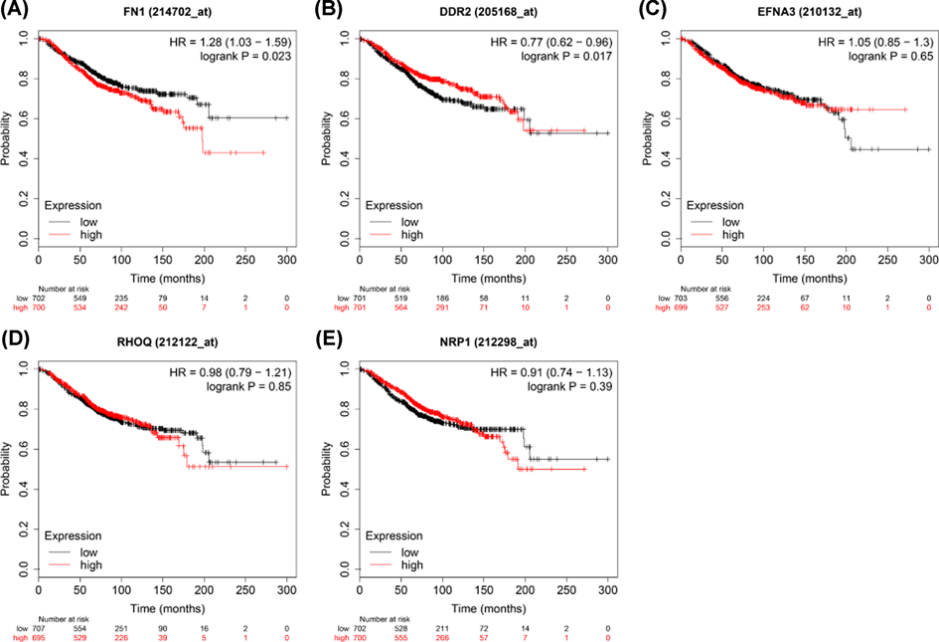
Overall survival analysis of 5 hub mrDEGs coregulated by TFs and miRNAs were performed by Kaplan–Meier plotter database (www.kmplot.com), which included 1402 breast cancer tissues Red color represents high expression and black color represents low expression. FN1 (**A**), DDR2 (**B**), EFNA3 (**C**), RHOQ (**D**), NRP1 (**E**); CI, confidence interval; DDR2, discoidin domain receptor tyrosine kinase 2; EFNA3, ephrin A3; FN1, fibronectin 1; HR, hazard ratio; mrDEGs, mitochondria-related differential expressed genes; NRP1, neuropilin 1; RHOQ, ras homolog family member Q; TFs, transcription factors.

Prognostic significance of the 5 coregulated hub mrDEGs by TFs and miRNAs in BC was evaluated using Kaplan–Meier plotter. Two hub mrDEGs with statistical significance were identified in survival analysis (*P*<0.05, *n*=1402) including FN1 (HR = 1.28 (1.03–1.59), *P*=0.023) and DDR2 (HR = 0.77 (0.62–0.96), *P*=0.017). Therefore, the up-regulated FN1 and down-regulated DDR2 might confer to poor BC prognosis (Figure 9).

## Discussion

In the present study, the microarray data of mitochondria-related genes in BC cell lines collected from GEO were utilized to identify mrDEGs and further validated in TCGA tissue samples. Moreover, GO enrichment analysis and KEGG pathway mapping for validated mrDEGs were performed to explore the potential function of mrDEGs in breast carcinogenesis. Based on that, we constructed PPI network, discovered and validated the hub mrDEGs. Furthermore, TF-miRNA-hub mrDEGs network was constructed and correlation of coregulated hub mrDEGs with the overall survival of BC patients was analyzed to investigate the influence of coregulated hub mrDEGs on BC prognosis.

Mitochondria function plays a critical role in multiple cell processes, and mitochondrial dysfunction may affect the occurrence and development of BC. The initiation and metastasis of BC could be altered by regulating the genetic background of mitochondria, making mrDEGs potential therapeutic targets [28]. Here, multiple bioinformatics tools were adopted to analyze the microarray data of mitochondria-related genes, and found out 23 up-regulated and 71 down-regulated mrDEGs in BC lines and tissue samples. They were closely associated with mitochondrial dysfunction in breast carcinogenesis. GO enrichment analysis demonstrated that 94 mrDEGs were enriched in cancer-related biological processes, such as neural crest cell migration involved in autonomic nervous system development, regulation of cell migration, cell surface receptor signaling pathway, cell differentiation and regulation of cell communication. These biological processes conformed to tumor cell properties, including unlimited cell proliferation, cell invasion and migration, and reduced intercellular adhesion [29], suggesting that mrDEGs were tightly linked to breast carcinogenesis. KEGG pathway mapping showed that mrDEGs might participate in cancer-related regulation pathways, including PI3K-ALT pathway, TGF-beta pathway, evading apoptosis and resistance to chemotherapy. Their relationship with BC could be listed as follows: (1) Inhibiting PI3K-AKT pathway may induce mitochondria-mediated cell apoptosis of BC [30]; (2) Ligand-dependent or cell-autonomous activation of the TGF-β pathway in stromal cells could induce metabolic reprogramming, enhance oxidative stress, mitochondrial autophagy and aerobic glycolysis, and decrease Cav-1, which can spread to adjacent fibroblasts and maintain BC cell growth [31]; (3) Regarding the well-known property of unlimited proliferation in BC cells, the GSTs gene mapped in evading apoptosis pathway could regulate cell apoptosis by its interaction with various protein partners [32]; (4) Melanocyte inducing transcription factor (MITF), a differential gene identified in our research, is able to enhance mitochondrial oxidative phosphorylation [33]. It has been reported that enhanced mitochondrial oxidative phosphorylation may induce the resistance to chemotherapy of BC cells [34]; thus, these genes could be related to drug resistance of BC cells. Overall, the validated mrDEGs mentioned above might be enriched in the pathways of BC progression through regulating mitochondrial function.

PPI network analysis indicated that three interaction networks could be classical models to predict BC occurrence. Model 1 consisted of SEMA3F, EFNA3, SEMA3A and NRP1, which were mainly associated with the structure and function of nerve cells. EFNA3 was induced by HIF under anoxic conditions, and then Ephrin-A3 protein encoded by EFNA3 was aberrantly accumulated to promote the metastasis of BC cells [35]. Model 2 was composed of BGN, DDR2 and COL5A2, which were mainly involved in extracellular matrix of cells. COL5A2 related to extracellular matrix remodeling was up-regulated during the development from ductal carcinoma *in situ* to invasive ductal carcinoma, leading to BC progression [36]. Model 3 included zinc finger E-box binding homeobox 1 (ZEB1), vimentin (VIM) and FN1, participating in tissue development and gene expression. ZEB1 increased the expression of vascular endothelial growth factor (VEGF) via paracrine to stimulate angiogenesis in BC [37]. ZEB1 also promoted epithelial–mesenchymal transformation (EMT), proliferation and migration of BC [38]. All the three models with different function took their parts in BC progression.

In our study, 9 hub mrDEGs were screened out based on MCC method, including up-regulated FN1, BGN, EFNA3, COL5A2 and SEMA3F as well as down-regulated RHOQ, SEMA3A, NRP1 and DDR2. Ualcan was utilized to validate and visualize the expression of 9 hub mrDEGs. TF-miRNA-hub mrDEGs network was constructed, which had important instruction significance to explore the potential regulatory mechanism of hub mrDEGs in BC. TF-miRNA-hub mrDEGs network showed that MAZ of TF nodes could interact with 3 hub mrDEGs including EFNA3, NRP1 and RHOQ, which implied its significance in BC. Myc-associated zinc finger protein (MAZ) has been considered as a transcription factor with C2H2 zinc finger motif binding to a GA box [39,40], with important roles in BC progression [40,41]. The study suggested that the transactivation and transcriptional alteration of MAZ could modulate the process of aerobic glycolysis in tumor [42]. NRP1 targeting hub mrDEGs of MAZ might be located in mitochondria regulating mitochondrial function and iron-dependent oxidative stress [43]. A GEO dataset (GSE115118) for miRNA mitochondrial sublocalization indicated that hsa-mir-218-5p, hsa-mir-26a-5p and hsa-mir-335-5p regulated by NRP1 were located in mitochondria. Therefore, MAZ was predicted to impact on mitochondria function by interacting with NRP1 and these miRNAs to regulate BC progression. Further investigation is needed to elucidate the function of these hub mrDEGs in BC.

Survival analysis showed that the up-regulated FN1 and down-regulated DDR2 suggested poor BC prognosis (*P*<0.05) with the potential to be a significant biomarker. FN1 has been demonstrated to be up-regulated in BC epithelial cells without mitochondria DNA [44]. FN1 was also a core gene of mrDEGs network and its encoded fibronection distributed in BC cell matrix affecting tumor progression [44]. It has been reported that FN1 can increase cisplatin resistance in non-small cell lung cancer by modulating β-catenin signaling via interaction with integrin-β1 [45]. FN1 might also be a potential biomarker for radioresistance in head and neck squamous cell carcinoma [46] and a potential therapeutic target for breast cancer [47]. We initially reported that FN1 was closely related to mitochondrial function. It had the highest degree score and could also interact with hsa-mir-218-5p and hsa-mir-26a-5p sublocated in mitochondria (GSE115118). Mitochondria are well accepted to exert crucial roles for anticancer drug target in tumor. Therefore, we could speculate that FN1 might be an anticancer drug target for BC by regulating mitochondrial dysfunction. DDR2 was activated by fibrillar collagen to regulate the synthesis of extracellular matrix and wound healing affecting microenvironment [48]. DDR2 was involved in hypoxia-induced cancer metastasis by accelerating migration, invasion and EMT of BC cells [49]. Our TF-miRNA-hub mrDEGs network showed that DDR2 could interact with hsa-mir-21-5p, hsa-mir-548a-3p and hsa-mir-129-2-3p, which were also sublocated in mitochondria (GSE115118). Further study for these genes would help to elucidate BC etiology from the perspective of mitochondrial dysfunction, and thus to identify diagnostic and prognostic biomarkers as well as molecular targets for BC targeted therapy.

In summary, bioinformatics analyses were employed to discover mrDEGs in BC. Gene enrichment analyses were carried out and three interaction networks were constructed to serve as classical models for predicting breast carcinogenesis. We also selected 5 coregulated hub mrDEGs by TFs and miRNAs including FN1, DDR2, NRP1, EFNA3 and RHOQ. And TF-miRNA-hub mrDEGs network was constructed to explore the potential pathogenesis of hub mrDEGs in BC.

## Supplementary Material

Supplementary Figure S1 and Tables S1-S2

## Data Availability

The data that support the results of this manuscript are available from the corresponding author upon reasonable request.

## Abbreviations

ANK2: Ankyrin 2
ARID4B: AT-rich interaction domain 4B
BC: breast cancer
BGN: biglycan
COL5A2: collagen type V alpha 2 chain
CXCL2: C-X-C motif chemokine ligand 2
DDR2: discoidin domain receptor tyrosine kinase 2
EFNA3: ephrin A3
FN1: fibronectin 1
GO: Gene Ontology
GPC2: Glypican 2
HDGF: heparin binding growth factor
KEGG: Kyoto Encyclopedia of Genes and Genomes
KLF16: Kruppel-like factor 16
KLF9: Kruppel-like factor 9
MARK1: microtubule affinity regulating kinase 1
MAZ: MYC associated zinc finger protein
MGST1: microsomal glutathione S-transferase 1
MITF: melanocyte inducing transcription factor
mrDEGs: mitochondria-related differential expressed genes
MXD4: MAX dimerization protein 4
NRP1: neuropilin 1
PIK3R1: phosphoinositide-3-kinase regulatory subunit 1
PPI: protein–protein network
RHOQ: ras homolog family member Q
SEMA3A: semaphorin 3A
SEMA3F: semaphorin 3F
SP2: Sp2 transcription factor
TF: transcription factor
VIM: vimentin
ZEB1: zinc finger E-box binding homeobox 1
