# Supplementary material for: Mitochondria-related core genes and TF-miRNA-hub mrDEGs network in breast cancer
Source: Biosci Rep. 2021 Jan 27;41(1):BSR20203481. doi: 10.1042/BSR20203481 (PMC7843495; doi:10.1042/BSR20203481)
Supplement: Supplementary Figure S1 and Tables S1-S2 [file BSR-2020-3481_supp.pdf]

Supplementary materials

Mitochondria-related core genesand TF-miRNA-hub mrDEGs network in breast cancer

Li-rong Yan[1], Ang Wang[1], Zhi Lv[1], Yuan Yuan[1]*, Qian Xu[1]**

[1]Tumor Etiology and Screening Department of Cancer Institute and General Surgery, The First Affiliated Hospital of China Medical University, Key Laboratory of Cancer Etiology and Prevention, China Medical University, Liaoning Provincial Education Department, Shenyang 110001, China.

****Corresponding author:** Dr. Qian Xu, Tumor Etiology and Screening Department of Cancer Institute and General Surgery, North Nanjing Street 155#, Heping District, Shenyang110001, China Telephone：+86-024-83282153; fax: +86-024-83282383. Email：qxu@cmu.edu.cn.

***Corresponding author:** Dr. Yuan Yuan, Tumor Etiology and Screening Department of Cancer Institute and General Surgery, North Nanjing Street 155#, HepingDistrict, Shenyang110001, China

Telephone：+86-024-83282153; fax: +86-024-83282383. Email：yuanyuan@cmu.edu.cn

Content:

Table S1 GO enrichment analysis of mitochondria-related differential expressed genes

Term ID	Term description	FDR	Genes
GO:0021637	trigeminal nerve structural organization	0.0027	NRP1,SEMA3A,SEMA3F
GO:0036486	ventral trunk neural crest cell migration	0.0027	NRP1,SEMA3A,SEMA3F
GO:0097490	sympathetic neuron projection extension	0.0027	NRP1,SEMA3A,SEMA3F
GO:0097491	sympathetic neuron projection guidance	0.0027	NRP1,SEMA3A,SEMA3F
GO:1902287	semaphorin-plexin signaling pathway involved in axon guidance	0.0027	NRP1,SEMA3A,SEMA3F
GO:0021612	facial nerve structural organization	0.0029	NRP1,SEMA3A,SEMA3F
GO:0021785	branchiomotor neuron axon guidance	0.0029	NRP1,SEMA3A,SEMA3F
GO:0001764	neuron migration	0.0033	ASPM,CELSR1,MARK1,MEF2C,NRP1,SEMA3A
GO:0014033	neural crest cell differentiation	0.0033	FN1,MEF2C,NRP1,SEMA3A,SEMA3F
GO:0040012	regulation of locomotion	0.0033	CXCL2,DDR2,DLC1,EPB41L4B,FN1,LIMCH1,MCTP1,MEF2C,MYLK,NRP1, PIK3R1,RECK,SEMA3A,SEMA3F,SNCA
GO:0048513	animal organ development	0.0033	ANK2,ASPM,BHLHE41,BICC1,CELSR1,COL5A2,COL9A3,CPS1,DDR2,DLC1, DOCK11,FN1,KRT81,LPIN1,LSR,MECOM,MEF2C,MGST1,MITF,MYLK,NFIB,NRP1, PIK3R1,PYGO1,SATB1,SEMA3A,SEMA3F,TACC1,VIM,ZEB1
GO:0048731	system development	0.0033	ANK2,ASPM,BHLHE41,BICC1,CELSR1,COL5A2,COL9A3,CPS1,DDR2,DLC1,DOCK11, EFNA3,FAM126A,FN1,GPC2,HES4,KRT81,LPIN1,LSR,MARK1,MECOM,MEF2C,MGST1, MITF,MYLK,NAP1L2,NFIB,NRP1,PIK3R1,PYGO1,QKI,RECK,SATB1,SEMA3A,SEMA3F, TACC1,VIM,ZEB1
GO:0048843	negative regulation of axon extension involved in axon guidance	0.0033	NRP1,SEMA3A,SEMA3F
GO:0048846	axon extension involved in axon guidance	0.0033	NRP1,SEMA3A,SEMA3F
GO:0051270	regulation of cellular component movement	0.0033	ANK2,CXCL2,DDR2,DLC1,EPB41L4B,FN1,LIMCH1,MCTP1,MEF2C,MYLK,NRP1,PIK3R1, RECK,SEMA3A,SEMA3F
GO:0060600	dichotomous subdivision of an epithelial terminal unit	0.0033	CELSR1,NRP1,SEMA3A
GO:0061549	sympathetic ganglion development	0.0033	NRP1,SEMA3A,SEMA3F

GO:0016043	cellular component organization	0.0039	ANK2,ASPM,BGN,CCDC136,COL5A2,COL9A3,DDR2,DLC1,EFNA3,EHHADH,EMP1, EPB41L4B,FGD4,FN1,H2AFJ,JADE1,KCTD12,LIMCH1,LPIN1,LSR,MAP7D3,MARK1, MECOM,MEF2C,MGST1,MITF,MYLK,NAP1L2,NFIB,NR3C1,NRP1,PADI3,PIK3R1,PYGO1, RECK,RHOQ,SATB1,SEMA3A,SEMA3F,SNCA,SYNM,TACC1,VIM
GO:0051271	negative regulation of cellular component movement	0.0046	DLC1,LIMCH1,MCTP1,MEF2C,NRP1,RECK,SEMA3A,SEMA3F
GO:0110020	regulation of actomyosin structure organization	0.0056	DLC1,LIMCH1,MEF2C,NRP1,PIK3R1
GO:0040013	negative regulation of locomotion	0.0058	DLC1,LIMCH1,MCTP1,MEF2C,NRP1,RECK,SEMA3A,SEMA3F
GO:0071417	cellular response to organonitrogen compound	0.0075	CACNA2D1,COL5A2,CPS1,LPIN1,MEF2C,PIK3R1,RHOQ,SNCA,ZEB1,ZNF106
GO:0021828	gonadotrophin-releasing hormone neuronal migration to the hypothalamus	0.0076	NRP1,SEMA3A
GO:0045664	regulation of neuron differentiation	0.0076	ASPM,EFNA3,FN1,MARK1,MEF2C,NAP1L2,NRP1,SEMA3A,SEMA3F,VIM,ZEB1
GO:0051960	regulation of nervous system development	0.0076	ASPM,CELSR1,EFNA3,FN1,MARK1,MEF2C,NAP1L2,NRP1,SEMA3A,SEMA3F,SNCA,VIM, ZEB1
GO:1903375	facioacoustic ganglion development	0.0076	NRP1,SEMA3A
GO:0007275	multicellular organism development	0.0077	ANK2,ASPM,BHLHE41,BICC1,CELSR1,COL5A2,COL9A3,CPS1,DDR2,DLC1,DOCK11, EFNA3,EMP1,FAM126A,FN1,GPC2,HES4,KRT81,LPIN1,LSR,MARK1,MECOM,MEF2C, MGST1,MITF,MYLK,NAP1L2,NFIB,NRP1,PIK3R1,PYGO1,QKI,RECK,SATB1,SEMA3A, SEMA3F,TACC1,VIM,ZEB1
GO:0150020	basal dendrite arborization	0.01	NRP1,SEMA3A
GO:0006928	movement of cell or subcellular component	0.0105	ANK2,ASPM,CACNA2D1,CELSR1,CXCL2,DPP4,EFNA3,FN1,MARK1,MEF2C,NFIB, NRP1,PIK3R1,PROS1,SEMA3A,SEMA3F,VIM
GO:0032970	regulation of actin filament-based process	0.0106	ANK2,CELSR1,DLC1,LIMCH1,MEF2C,NRP1,PIK3R1,RHOQ

GO:0032501	multicellular organismal process	0.0112	ANK2,ASPM,BGN,BHLHE41,BICC1,CACNA2D1,CCDC136,CCDC50,CELSR1,COL5A2,COL9A3,CPS1,DDR2,DLC1,DOCK11,DPP4,EFNA3,EMP1,FAM126A,FN1,GPAM,GPC2,HES4,KRT81,LPIN1,LSR,MARK1,MECOM,MEF2C,MGST1,MITF,MYLK,NAP1L2,NFIB,NRP1,PIK3R1,PROS1,PYGO1,QKI,RECK,SATB1,SEMA3A,SEMA3F,SNCA,SYNM,TACC1,VIM,ZEB1
GO:0032502	developmental process	0.0113	ANK2,ARHGEF28,ASPM,BHLHE41,BICC1,CCDC136,CELSR1,COL5A2,COL9A3,CPS1,DDR2,DLC1,DOCK11,EFNA3,EMP1,FAM126A,FN1,GPC2,HES4,KRT81,LPIN1,LSR,MARK1,MECOM,MEF2C,MGST1,MITF,MYLK,NAP1L2,NFIB,NRP1,PIK3R1,PYGO1,QKI,RECK,SATB1,SEMA3A,SEMA3F,SNCA,TACC1,VIM,ZEB1
GO:0061551	trigeminal ganglion development	0.0122	NRP1,SEMA3A
GO:0023051	regulation of signaling	0.0128	AKAP12,ANK2,ARHGEF28,ARHGEF40,ASPM,BGN,BICC1,CXCL2,DEPDC7,DLC1,DPP4,FAM132A,FGD4,FN1,GPAM,GPC2,JADE1,MAP3K8,MCTP1,MDFIC,MECOM,MEF2C,NRP1,PIK3R1,PIP5K1B,RHOQ,SEMA3A,SEMA3F,SNCA,ZEB1
GO:0048856	anatomical structure development	0.0128	ANK2,ASPM,BHLHE41,BICC1,CCDC136,CELSR1,COL5A2,COL9A3,CPS1,DDR2,DLC1,DOCK11,EFNA3,EMP1,FAM126A,FN1,GPC2,HES4,KRT81,LPIN1,LSR,MARK1,MECOM,MEF2C,MGST1,MITF,MYLK,NAP1L2,NFIB,NRP1,PIK3R1,PYGO1,QKI,RECK,SATB1,SEMA3A,SEMA3F,TACC1,VIM,ZEB1
GO:0050793	regulation of developmental process	0.013	ASPM,BHLHE41,CELSR1,COL5A2,DDR2,DLC1,EFNA3,FGD4,FN1,GPAM,ISG15,MARK1,MEF2C,MITF,NAP1L2,NFIB,NRP1,PIK3R1,RHOQ,SEMA3A,SEMA3F,SNCA,VIM,ZEB1
GO:0034764	positive regulation of transmembrane transport	0.0131	ANK2,CACNA2D1,FAM132A,PIK3R1,RHOQ,SNCA
GO:0007435	salivary gland morphogenesis	0.0141	NFIB,NRP1,SEMA3A
GO:0035023	regulation of Rho protein signal transduction	0.0148	ARHGEF28,ARHGEF40,DLC1,FGD4,NRP1
GO:0051491	positive regulation of filopodium assembly	0.0148	DOCK11,NRP1,RHOQ
GO:0060666	dichotomous subdivision of terminal units involved in salivary gland branching	0.0148	NRP1,SEMA3A

GO:0016477	cell migration	0.0159	ASPM,CELSR1,CXCL2,DPP4,FN1,MARK1,MEF2C,NRP1,PIK3R1,PROS1,SEMA3A,SEMA3F
GO:0032956	regulation of actin cytoskeleton organization	0.0175	CELSR1,DLC1,LIMCH1,MEF2C,NRP1,PIK3R1,RHOQ
GO:0051492	regulation of stress fiber assembly	0.0175	DLC1,LIMCH1,NRP1,PIK3R1
GO:0060385	axonogenesis involved in innervation	0.0175	NRP1,SEMA3A
GO:0007010	cytoskeleton organization	0.0179	ANK2,ASPM,DLC1,EPB41L4B,FGD4,LIMCH1,MAP7D3,MARK1,NRP1,RHOQ,SYNM,TACC1, VIM
GO:0030029	actin filament-based process	0.0179	ANK2,CACNA2D1,DLC1,EPB41L4B,FGD4,LIMCH1,NRP1,RHOQ,VIM
GO:0007411	axon guidance	0.0198	EFNA3,NFIB,NRP1,PIK3R1,SEMA3A,SEMA3F
GO:0006644	phospholipid metabolic process	0.02	FAM126A,GPAM,ISYNA1,LPIN1,PIK3R1,PIP5K1B,SERINC2,SNCA
GO:0030516	regulation of axon extension	0.02	FN1,NRP1,SEMA3A,SEMA3F
GO:0001667	ameboidal-type cell migration	0.0201	DPP4,FN1,NRP1,SEMA3A,SEMA3F
GO:0051056	regulation of small GTPase mediated signal transduction	0.0201	ARHGEF28,ARHGEF40,DEPDC7,DLC1,FGD4,NRP1,RHOQ
GO:0009966	regulation of signal transduction	0.0206	AKAP12,ARHGEF28,ARHGEF40,ASPM,BGN,BICC1,CXCL2,DEPDC7,DLC1,FAM132A, FGD4,FN1,GPAM,GPC2,JADE1,MAP3K8,MDFIC,MECOM,MEF2C,NRP1,PIK3R1,PIP5K1B, RHOQ,SEMA3A,SEMA3F,SNCA,ZEB1
GO:0006638	neutral lipid metabolic process	0.0217	CPS1,GPAM,LPIN1,SNCA
GO:0009987	cellular process	0.0241	AKAP12,ANK2,ARHGEF28,ASPM,BGN,BHLHE41,CACNA2D1,CCDC136,CELSR1,CHTF18 ,COL5A2,COL9A3,CPS1,CXCL2,DDR2,DEPDC7,DLC1,DOCK11,DPP4,DSEL,EFNA3,EHHADH, EMP1,EPB41L4B,FAM126A,FGD4,FIGN,FN1,GPAM,GPC2,H2AFJ,HES4,ISG15,ISYNA1,JADE1, KCTD12,KRT81,LIMCH1,LPIN1,LRCH2,LSR,MAP3K8,MAP7D3,MARK1,MBNL2,MCTP1, MDFIC,MECOM,MEF2C,MGST1,MITF,MSRB3,MYLK,NAP1L2,NFIB,NR3C1,NR3C2,NRP1, NXPH4,OASL,PADI3,PARD3B,PIK3R1,PIP5K1B,PROS1,PTPN21,PYGO1,QKI,RECK,RHOQ, SACS,SATB1,SEMA3A,SEMA3F,SERINC2,SNCA,SYNM,TACC1,VIM,VKORC1L1,ZDHHC2, ZEB1,ZNF106

GO:0010604	positive regulation of macromolecule metabolic process	0.0246	ANK2,CHTF18,DDR2,DLC1,EFNA3,EPB41L4B,FN1,JADE1,LIMCH1,LPIN1,MAP3K8,MDFIC, MECOM,MEF2C,MITF,NAP1L2,NFIB,NR3C1,NRP1,PIK3R1,PYGO1,QKI,RHOQ,SEMA3A, SNCA,VIM,ZEB1
GO:0030336	negative regulation of cell migration	0.0248	DLC1,LIMCH1,MCTP1,MEF2C,RECK,SEMA3A
GO:0040011	locomotion	0.0262	ASPM,CELSR1,CXCL2,DPP4,EFNA3,FN1,MARK1,MEF2C,NFIB,NRP1,PIK3R1,PROS1, SEMA3A,SEMA3F
GO:0051128	regulation of cellular component organization	0.0267	CELSR1,DDR2,DLC1,DOCK11,DPP4,FGD4,FN1,JADE1,LIMCH1,MARK1,MCTP1, MEF2C,MITF,NAP1L2,NRP1,PIK3R1,RHOQ,SACS,SEMA3A,SEMA3F,SNCA,VIM
GO:1901701	cellular response to oxygen-containing compound	0.0278	CACNA2D1,COL5A2,CPS1,LPIN1,MEF2C,MGST1,NR3C1,PIK3R1,RHOQ,SNCA,ZEB1,ZNF106
GO:0046326	positive regulation of glucose import	0.0293	FAM132A,PIK3R1,RHOQ
GO:2000026	regulation of multicellular organismal development	0.0298	ASPM,CELSR1,COL5A2,DDR2,EFNA3,FN1,ISG15,MARK1,MEF2C,MITF,NAP1L2,NFIB, NRP1,PIK3R1,SEMA3A,SEMA3F,SNCA,VIM,ZEB1
GO:0032060	bleb assembly	0.0301	EMP1,MYLK
GO:0071679	commissural neuron axon guidance	0.0301	NFIB,NRP1
GO:0048522	positive regulation of cellular process	0.0315	AKAP12,ANK2,ASPM,CHTF18,CXCL2,DDR2,DLC1,DOCK11,DPP4,EFNA3,EPB41L4B, FAM132A,FGD4,FN1,GPAM,ISG15,JADE1,LIMCH1,LPIN1,MAP3K8,MDFIC,MECOM, MEF2C,MITF,MYLK,NAP1L2,NFIB,NR3C1,NRP1,PIK3R1,PYGO1,QKI,RHOQ,SEMA3A, SNCA,VIM,ZEB1
GO:0030335	positive regulation of cell migration	0.032	CXCL2,DDR2,EPB41L4B,FN1,MYLK,NRP1,PIK3R1,SEMA3A
GO:0007399	nervous system development	0.0324	ANK2,ASPM,CELSR1,DLC1,EFNA3,FAM126A,FN1,GPC2,HES4,MARK1,MEF2C,NAP1L2, NFIB,NRP1,PIK3R1,QKI,SEMA3A,SEMA3F,TACC1,VIM,ZEB1

GO:0007165	signal transduction	0.0331	AKAP12,ANK2,ARHGEF28,BGN,CELSR1,CXCL2,DDR2,DEPDC7,DLC1,DOCK11, EFNA3,FGD4,FN1,GPC2,ISG15,LRCH2,MAP3K8,MARK1,MCTP1,MDFIC,MEF2C,MITF, NR3C1,NR3C2,NRP1,NXPH4,OASL,PIK3R1,PYGO1,RHOQ,SEMA3A,SEMA3F,SNCA,VIM, ZEB1,ZNF106
GO:1902531	regulation of intracellular signal transduction	0.0339	AKAP12,ARHGEF28,ARHGEF40,BGN,DEPDC7,DLC1,FAM132A,FGD4,FN1,MAP3K8, MDFIC,MECOM,MEF2C,NRP1,PIK3R1,PIP5K1B,RHOQ,SEMA3A
GO:0001952	regulation of cell-matrix adhesion	0.0352	DLC1,LIMCH1,NRP1,PIK3R1
GO:0048583	regulation of response to stimulus	0.0362	AKAP12,ARHGEF28,ARHGEF40,ASPM,BGN,BICC1,CXCL2,DEPDC7,DLC1,FAM132A, FGD4,FIGN,FN1,GPAM,GPC2,JADE1,MAP3K8,MCTP1,MDFIC,MECOM,MEF2C,MYLK, NRP1,PIK3R1,PIP5K1B,PROS1,RHOQ,SEMA3A,SEMA3F,SNCA,ZEB1
GO:0030208	dermatan sulfate biosynthetic process	0.0369	BGN,DSEL
GO:0044255	cellular lipid metabolic process	0.037	CPS1,EHHADH,FAM126A,GPAM,GPC2,ISYNA1,LPIN1,PIK3R1,PIP5K1B,QKI,SERINC2,SNCA
GO:0021987	cerebral cortex development	0.0385	ASPM,NRP1,SEMA3A,TACC1
GO:0051493	regulation of cytoskeleton organization	0.0407	CELSR1,DLC1,LIMCH1,MEF2C,NRP1,PIK3R1,RHOQ,SNCA
GO:0022603	regulation of anatomical structure morphogenesis	0.0408	CELSR1,COL5A2,DLC1,EFNA3,FGD4,FN1,MEF2C,NFIB,NRP1,RHOQ,SEMA3A,SEMA3F
GO:0030205	dermatan sulfate metabolic process	0.0408	BGN,DSEL
GO:0031295	T cell costimulation	0.0408	DPP4,MAP3K8,PIK3R1
GO:0010810	regulation of cell-substrate adhesion	0.0413	DLC1,FN1,LIMCH1,NRP1,PIK3R1
GO:0045785	positive regulation of cell adhesion	0.0413	DPP4,EPB41L4B,FN1,GPAM,MAP3K8,NRP1,PIK3R1
GO:0051241	negative regulation of multicellular organismal process	0.0413	ASPM,COL5A2,EFNA3,FN1,ISG15,MEF2C,NFIB,NRP1,PIK3R1,PROS1,SEMA3A,SEMA3F,VIM
GO:0032989	cellular component morphogenesis	0.0427	ANK2,CCDC136,EFNA3,FN1,MEF2C,NFIB,NRP1,PIK3R1,SEMA3A,SEMA3F
GO:0051928	positive regulation of calcium ion transport	0.0427	ANK2,CACNA2D1,MYLK,SNCA
GO:0120035	regulation of plasma membrane bounded cell projection organization	0.0427	DOCK11,FN1,MARK1,MEF2C,NRP1,RHOQ,SEMA3A,SEMA3F,VIM

GO:0046928	regulation of neurotransmitter secretion	0.0442	MCTP1,MEF2C,SNCA
GO:0051173	positive regulation of nitrogen compound metabolic process	0.0447	CHTF18,DDR2,DLC1,EFNA3,FN1,JADE1,LIMCH1,LPIN1,MAP3K8,MDFIC,MECOM, MEF2C,MITF,NAP1L2,NFIB,NR3C1,NRP1,PIK3R1,PYGO1,QKI,RHOQ,SEMA3A,SNCA, VIM,ZEB1
GO:0000904	cell morphogenesis involved in differentiation	0.0453	EFNA3,FN1,MEF2C,NFIB,NRP1,PIK3R1,SEMA3A,SEMA3F
GO:0010715	regulation of extracellular matrix disassembly	0.0453	DDR2,DPP4
GO:0035239	tube morphogenesis	0.0453	CELSR1,DLC1,FN1,MEF2C,MYLK,NFIB,NRP1,QKI,ZEB1
GO:0045595	regulation of cell differentiation	0.0453	ASPM,BHLHE41,COL5A2,DDR2,EFNA3,FN1,ISG15,MARK1,MEF2C,MITF,NAP1L2, NRP1,PIK3R1,SEMA3A,SEMA3F,VIM,ZEB1
GO:0051239	regulation of multicellular organismal process	0.0453	ANK2,ASPM,CACNA2D1,CELSR1,COL5A2,DDR2,EFNA3,EPB41L4B,FN1,GPAM,ISG15, MARK1,MEF2C,MITF,NAP1L2,NFIB,NRP1,PIK3R1,PROS1,SEMA3A,SEMA3F,SNCA, VIM,ZEB1
GO:0051893	regulation of focal adhesion assembly	0.0453	DLC1,LIMCH1,NRP1
GO:1901653	cellular response to peptide	0.0453	CACNA2D1,CPS1,LPIN1,PIK3R1,RHOQ,ZNF106
GO:0003012	muscle system process	0.0455	ANK2,CACNA2D1,MEF2C,MYLK,SYNM,VIM
GO:0051716	cellular response to stimulus	0.0475	AKAP12,ANK2,ARHGEF28,BGN,CACNA2D1,CELSR1,COL5A2,CPS1,CXCL2,DDR2, DEPDC7,DLC1,DOCK11,EFNA3,FGD4,FN1,GPC2,ISG15,LPIN1,LRCH2,MAP3K8, MARK1,MCTP1,MDFIC,MEF2C,MGST1,MITF,MYLK,NR3C1,NR3C2,NRP1,NXPH4, OASL,PIK3R1,PYGO1,RHOQ,SEMA3A,SEMA3F,SNCA,VIM,VKORC1L1,ZEB1,ZNF106
GO:0010243	response to organonitrogen compound	0.048	CACNA2D1,COL5A2,CPS1,LPIN1,MEF2C,MGST1,PIK3R1,RHOQ,SNCA,ZEB1,ZNF106
GO:0023052	signaling	0.048	AKAP12,ANK2,ARHGEF28,BGN,CACNA2D1,CELSR1,CXCL2,DDR2,DEPDC7, DLC1,DOCK11,EFNA3,FGD4,FN1,GPC2,ISG15,LRCH2,MAP3K8,MARK1,MCTP1, MDFIC,MEF2C,MITF,NR3C1,NR3C2,NRP1,NXPH4,OASL,PIK3R1,PYGO1,RHOQ, SEMA3A,SEMA3F,SNCA,VIM,ZEB1,ZNF106
GO:0030155	regulation of cell adhesion	0.048	DLC1,DPP4,EPB41L4B,FN1,GPAM,LIMCH1,MAP3K8,NRP1,PIK3R1

GO:0030198	extracellular matrix organization	0.048	BGN,COL5A2,COL9A3,DDR2,FN1,RECK
GO:0045665	negative regulation of neuron differentiation	0.048	ASPM,NRP1,SEMA3A,SEMA3F,VIM
GO:0046486	glycerolipid metabolic process	0.048	CPS1,FAM126A,GPAM,LPIN1,PIK3R1,PIP5K1B,SERINC2
GO:0048667	cell morphogenesis involved in neuron differentiation	0.048	EFNA3,MEF2C,NFIB,NRP1,PIK3R1,SEMA3A,SEMA3F
GO:0048699	generation of neurons	0.048	ASPM,CELSR1,EFNA3,FN1,GPC2,MARK1,MEF2C,NAP1L2,NFIB,NRP1, PIK3R1,SEMA3A,SEMA3F,VIM,ZEB1
GO:0065009	regulation of molecular function	0.048	ANK2,ARHGEF28,ARHGEF40,BGN,CACNA2D1,CHTF18,CXCL2,DDR2, DEPDC7,DLC1,DOCK11,EFNA3,FAM132A,FGD4,FN1,MAP3K8,MDFIC,MEF2C, NFIB,NRP1,PIK3R1,PROS1,RECK,SEMA3A,SEMA3F,SERINC2,SNCA
GO:0051129	negative regulation of cellular component organization	0.0489	DLC1,DPP4,MCTP1,NRP1,SACS,SEMA3A,SEMA3F,SNCA,VIM
GO:0048638	regulation of developmental growth	0.0494	FN1,GPAM,MEF2C,NRP1,SEMA3A,SEMA3F
GO:0030318	melanocyte differentiation	0.0498	MEF2C,MITF
GO:0086012	membrane depolarization during cardiac musclecell action	0.0498	ANK2,CACNA2D1

Table S2 Enrichment analysis of Modules

Category	Term ID	Term description	FDR	Genes
Module 1	GO:0048843	negative regulation of axon extension involved in axon guidance	3.66E-08	NRP1,SEMA3A,SEMA3F
	GO:0048846	axon extension involved in axon guidance	3.66E-08	NRP1,SEMA3A,SEMA3F
	GO:0061549	sympathetic ganglion development	3.66E-08	NRP1,SEMA3A,SEMA3F
	GO:0097490	sympathetic neuron projection extension	3.66E-08	NRP1,SEMA3A,SEMA3F
	GO:0097491	sympathetic neuron projection guidance	3.66E-08	NRP1,SEMA3A,SEMA3F
	GO:1901166	neural crest cell migration involved in autonomic nervous system development	3.66E-08	NRP1,SEMA3A,SEMA3F
	GO:1902287	semaphorin-plexin signaling pathway involved in axon guidance	3.66E-08	NRP1,SEMA3A,SEMA3F
	GO:0007411	axon guidance	2.81E-07	EFNA3,NRP1,SEMA3A,SEMA3F
	GO:0021828	gonadotrophin-releasing hormone neuronal migration to the hypothalamus	2.10E-06	NRP1,SEMA3A
	GO:1903375	facioacoustic ganglion development	2.10E-06	NRP1,SEMA3A
	GO:0150020	basal dendrite arborization	2.93E-06	NRP1,SEMA3A

	GO:0061551	trigeminal ganglion development	3.68E-06	NRP1,SEMA3A
	GO:0060666	dichotomous subdivision of terminal units involved in salivary gland branching	4.82E-06	NRP1,SEMA3A
	GO:0045664	regulation of neuron differentiation	6.05E-06	EFNA3,NRP1,SEMA3A,SEMA3F
	GO:0060385	axonogenesis involved in innervation	6.05E-06	NRP1,SEMA3A
	GO:0051093	negative regulation of developmental process	2.55E-05	EFNA3,NRP1,SEMA3A,SEMA3F
	GO:0022603	regulation of anatomical structure morphogenesis	3.08E-05	EFNA3,NRP1,SEMA3A,SEMA3F
	GO:0007413	axonal fasciculation	4.05E-05	NRP1,SEMA3A
	GO:0051241	negative regulation of multicellular organismal process	4.84E-05	EFNA3,NRP1,SEMA3A,SEMA3F
	GO:0050919	negative chemotaxis	8.87E-05	SEMA3A,SEMA3F
	GO:0010469	regulation of signaling receptor activity	0.00037	NRP1,SEMA3A,SEMA3F
	GO:0007166	cell surface receptor signaling pathway	0.00055	EFNA3,NRP1,SEMA3A,SEMA3F
	GO:0018108	peptidyl-tyrosine phosphorylation	0.0018	EFNA3,NRP1
	GO:0010632	regulation of epithelial cell migration	0.0021	NRP1,SEMA3A
	GO:0065009	regulation of molecular function	0.0025	EFNA3,NRP1,SEMA3A,SEMA3F
	GO:0045765	regulation of angiogenesis	0.0035	EFNA3,NRP1
	GO:0032270	positive regulation of cellular protein metabolic process	0.0049	EFNA3,NRP1,SEMA3A
	GO:0048880	sensory system development	0.0053	NRP1,SEMA3A
	GO:0050769	positive regulation of neurogenesis	0.0077	NRP1,SEMA3A
	GO:0030335	positive regulation of cell migration	0.0086	NRP1,SEMA3A
	GO:0007169	transmembrane receptor protein tyrosine kinase signaling pathway	0.0101	EFNA3,NRP1
	GO:0043410	positive regulation of MAPK cascade	0.0105	NRP1,SEMA3A
	GO:0051345	positive regulation of hydrolase activity	0.0203	EFNA3,NRP1
	GO:0007267	cell-cell signaling	0.0378	EFNA3,NRP1
Module 3	GO:0043010	camera-type eye development	0.0253	VIM,ZEB1
	GO:0010975	regulation of neuron projection development	0.0316	FN1,VIM
	GO:0002376	immune system process	0.0317	FN1,VIM,ZEB1
	GO:0048598	embryonic morphogenesis	0.0338	FN1,ZEB1
	GO:0035239	tube morphogenesis	0.0382	FN1,ZEB1
	GO:0044403	symbiont process	0.0386	FN1,VIM

GO:0048513	animal organ development	0.0387	FN1,VIM,ZEB1
GO:0051173	positive regulation of nitrogen compound metabolic process	0.0387	FN1,VIM,ZEB1
GO:0045596	negative regulation of cell differentiation	0.0393	VIM,ZEB1
GO:0031325	positive regulation of cellular metabolic process	0.0409	FN1,VIM,ZEB1
GO:0008022	protein C-terminus binding	0.0181	FN1,VIM
hsa05206	MicroRNAs in cancer	0.00071	VIM,ZEB1

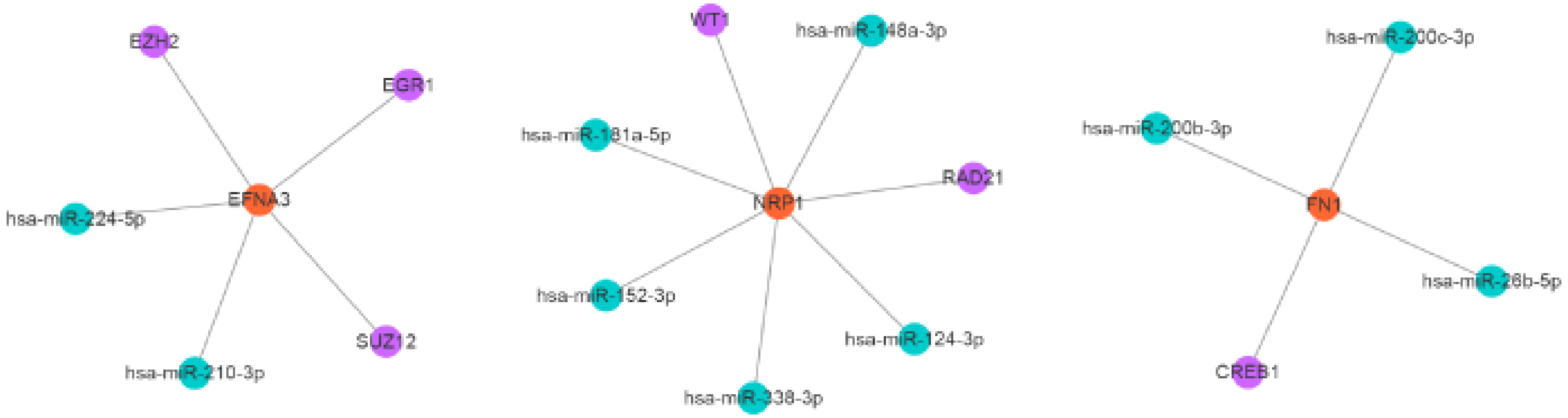

Figure S1: Validation of TF-miRNA-Hub mrDEGs network with starBase database (http://starbase.sysu.edu.cn/starbase2/index.php) and ChEA database with NetworkAnalyst (https://www.networkanalyst.ca/). Red color represented mrDEGs. Blue color represented microRNA and purple color represented TF.mrDEGs, mitochondria-related differential expressed genes; TF, transcription factor.
